# Eosinophiles anuläres Erythem bei einem 20 Monate alten Mädchen

**DOI:** 10.1007/s00105-020-04687-z

**Published:** 2020-09-15

**Authors:** Verena Paulitschke, Julia Tittes, Adrian Tanew, Sonja Radakovic

**Affiliations:** Universitätsklinik für Dermatologie, Medizinische Universität Wien, Währinger Gürtel 18–20, 1090 Wien, Österreich

**Keywords:** Eosinophile Dermatose, Wells-Syndrom, Histologie, Urtikarielles Entzündungsmuster, Urtikarielle Läsionen, Eosinophilic dermatosis, Wells syndrome, Histology, Urticarial inflammation pattern, Urticarial lesions

## Abstract

Wir berichten über ein 20 Monate altes Mädchen mit teils anulären, urtikariellen Hautveränderungen am gesamten Integument, die bis zu 1 Woche persistierten und dann an neuen Stellen auftraten. Die histologische Aufarbeitung zeigte ein urtikarielles Entzündungsmuster mit interstitiellem Ödem und einem Infiltrat mit Lymphozyten, Neutrophilen und vielen eosinophilen Granulozyten ohne Flammenfiguren. Das Labor war unauffällig. In Zusammenschau stellten wir die Diagnose eines eosinophilen anulären Erythems (EAE) im Kindesalter, das zu den eosinophilen Dermatosen zählt.

## Abstract (en)

We report on a 20-month-old girl with urticarial and partially annular skin lesions that were disseminated over the whole integument. The lesions persisted over 1 week and then gradually faded and reappeared on new body sites. The histological examination of a skin biopsy revealed an urticarial inflammation pattern with interstitial edema and a diffuse infiltration with many eosinophilic granulocytes without flame figures, neutrophils and lymphocytes. Laboratory investigations were inconspicuous and there was no eosinophilia. A diagnosis of eosinophilic annular erythema (EAE) of childhood was made which is a benign self-limiting skin disorder belonging to the group of eosinophilic dermatoses.

## Falldarstellung

### Anamnese und klinischer Befund

**Figure 1:**
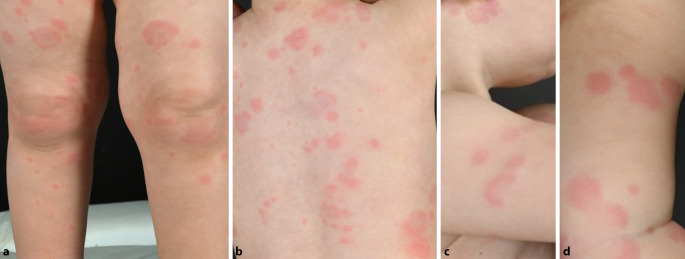

Wir berichten über ein 20 Monate altes Mädchen mit teils anulären, targetoiden, urtikariellen Hautveränderungen am gesamten Integument inklusive der Kopfhaut, die seit 6 Monaten bestanden und 3 Wochen nach einer Masern-Mumps-Röteln-Impfung aufgetreten waren (Abb. 1a–d). Die einzelnen Läsionen waren bis zu 5 cm groß und zeigten stellenweise eine zentrale Aufhellung. Sie persistierten bis zu 1 Woche und traten dann an neuen Stellen auf. Es bestand leichter Juckreiz. Das Mädchen war ansonsten völlig gesund und hatte keine nennenswerten Vorerkrankungen. Differenzialdiagnostisch dachten wir an ein Wells-Syndrom, eine Urtikariavaskulitis, ein Erythema anulare centrifugum und ein eosinophiles anuläres Erythem.

### Histopathologie und Labordiagnostik

**Figure 2:**
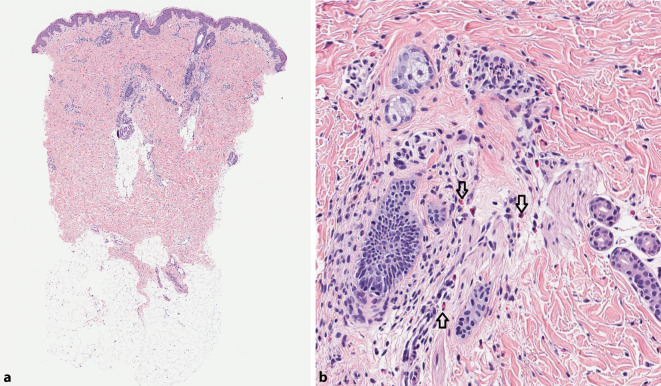

Die histologische Aufarbeitung eines Hautbiopsats vom Rücken zeigte ein urtikarielles Entzündungsmuster mit interstitiellem Ödem und einem Infiltrat mit Lymphozyten, vielen eosinophilen Granulozyten und Neutrophilen, das über die gesamte Breite der Dermis verteilt war. Flammenfiguren oder eine basale Melaninhyperpigmentierung waren nicht vorhanden (Abb. 2a, b). Die direkte Immunfluoreszenz war negativ. Blutbild, Differenzialblutbild, Serumchemie, antinukleäre Antikörper, ENA (extrahierbare nukleäre Antigene), allergologische Parameter (Gesamt-Ig[Immunglobulin]E, Antikörper gegen Inhalations- und Nahrungsmittelallergene), Immunglobuline und Elektrophorese waren unauffällig.

### Diagnose

In Zusammenschau von Anamnese, Klinik, Histopathologie und Labor stellten wir die Diagnose eines eosinophilen anulären Erythems (EAE) im Kindesalter.

### Therapie und Verlauf

Symptomatisch erhielt die kleine Patientin kurzzeitig ein Antihistaminikum, das den Juckreiz reduzierte. Bei der letzten Kontrolluntersuchung an unserer Klinik 3 Monate später war lediglich ein leichter Rückgang der Krankheitsaktivität zu erkennen.

## Diskussion

Das EAE zählt zu den seltenen, eosinophilen Dermatosen und wurde erstmals von Peterson und Jarratt 1981 [8] beschrieben. Es wurde anfangs als eine Erkrankung des Kindesalters angesehen und als „anuläres Erythem des Kindesalters“ bezeichnet [6, 8], in der Zwischenzeit liegen jedoch auch Fallberichte über das Auftreten von EAE bei Erwachsenen vor [5, 7]. Klinisch ist das EAE durch anuläre, erythematöse oder urtikarielle Läsionen mit zentrifugalem Wachstum charakterisiert, die teilweise zentral livid-bräunlich imponieren. Bei Progredienz kann es zur Ausbildung von gyrierten Erythemen mit zentraler Abblassung kommen, die Tage bis Wochen persistieren. Die Prädilektionsstellen sind der Stamm und die oberen Extremitäten, es kann aber das gesamte Integument inklusive Kapillitium befallen sein [1, 6]. Die Erkrankung ist durch einen protrahiert schubhaften Verlauf über Monate bis Jahre gekennzeichnet. Histopathologisch zeigt sich in der Dermis ein dichtes Infiltrat mit Eosinophilen, wobei es im Gegensatz zum Wells-Syndrom nicht zur Ausbildung von Flammenfiguren kommt. Die laborchemische Untersuchung ist in der Regel unauffällig, insbesondere liegt typischerweise keine periphere Eosinophilie vor. Die Ätiologie ist unbekannt, in Einzelfällen wurde eine mögliche Assoziation mit Autoimmunthyreoiditis, Borreliose und Nierenzellkarzinom diskutiert [4, 5]. In einer rezenten japanischen Studie an 10 Erwachsenen mit EAE, von denen 9 klinisch eine Hyperpigmentierung im Zentrum der Läsionen aufwiesen, wurden als zusätzliches histologisches Kriterium des EAE eine vermehrte Melaninablagerung im Stratum basale sowie eine Pigmentinkontinenz gefunden [7]. Die Autoren vermuten, dass dabei Interleukin‑5 eine entscheidende Rolle spielen könne, da es für die Chemoattraktion von Eosinophilen zuständig ist und die Melanogenese in den Melanozyten stimuliert. Bei unserer kleinen Patientin konnten wir weder klinisch noch histopathologisch eine epidermale Hyperpigmentierung nachweisen.

Daten zur Therapie des EAE sind aufgrund der extremen Seltenheit der Erkrankung beschränkt. Topische und systemische Glukokortikoide und Antimalariamittel (Chloroquin oder Hydroxychloroquin), welche die Chemotaxis von Eosinophilen inhibieren, wurden mit unterschiedlichem Erfolg eingesetzt [1, 2, 4, 7]. In therapierefraktären Einzelfällen wurden Erfolge mit Mepolizumab, ein humanisierter monoklonaler Antikörper gegen Interleukin 5 [11], und Dupilumab, ein Interleukin-4-Rezeptor-α-Antagonist [3], erzielt. Aufgrund des selbstlimitierenden Erkrankungsverlaufes und der zumeist milden Symptomatik muss im Einzelfall entschieden werden, ob ein Therapieversuch indiziert ist.

### Differenzialdiagnosen

Als Differenzialdiagnosen des EAE kommen Wells-Syndrom, Urtikariavaskulitis und Erythema anulare centrifugum in Betracht. Das Wells-Syndrom präsentiert sich typischerweise in Form von teils großflächigen urtikariellen Erythemen oder indurierten Plaques, die mit Juckreiz, Brennen oder Schmerzen einhergehen können. Auch bullöse oder granulomatöse Manifestationen wurden beschrieben. Als Allgemeinsymptomatik können Fieber oder Arthralgien bestehen. Der Verlauf ist oft chronisch rezidivierend mit Spontanremission nach Monaten bis Jahren. Histopathologisch zeigt sich eine Gewebeeosinophilie, wobei das Infiltrat oft bis in die Subkutis reicht. Zusätzlich findet man die typischen, jedoch nicht pathognomonischen Flammenfiguren, die herdförmigen Ansammlungen von zerfallenden eosinophilen Granulozyten entsprechen. Des Weiteren ist das Wells-Syndrom im Gegensatz zum EAE durch eine periphere Eosinophilie gekennzeichnet [1, 4, 7]. Trotz der Unterscheidungskriterien wird von einigen Autoren postuliert, dass das EAE eine Variante des Wells-Syndroms darstellt [2, 9]*.*

Die Urtikariavaskulitis ist klinisch durch ein urtikarielles Exanthem gekennzeichnet, bei dem die Läsionen länger als 24 h persistieren, eine Purpurakomponente aufweisen und mit Hyperpigmentierung abheilen. Die Hautveränderungen können jucken oder schmerzen und sind oft mit Allgemeinsymptomen wie Fieberschüben, Arthralgien oder Abdominalbeschwerden assoziiert. Histologisch findet man eine Vaskulitis mit einem perivaskulären Infiltrat von vorwiegend neutrophilen Granulozyten und eine ausgeprägter Leukozytoklasie [10].

Das Erythema anulare centrifugum (EAC) manifestiert sich zumeist mit einer halskrausenartigen Schuppung, seltener Vesikel oder Krusten, entlang des inneren Randes von bogenförmig angeordneten Erythemen. Histologisch ist das EAC durch dichte, scharf abgegrenzte, perivaskulär angeordnete lymphozytäre Infiltrate mit fakultativer Beimengung von Eosinophilen und diskreten epidermalen Veränderungen mit Spongiose und Parakeratose charakterisiert.

Zusammenfassend handelt es sich bei EAE um eine harmlose, selbstlimitierte Erkrankung ungeklärter Ätiologie, die trotz ihrer Seltenheit differenzialdiagnostisch bei chronisch rezidivierenden figurierten Erythemen insbesondere im Kindesalter berücksichtigt werden sollte.

## Fazit für die Praxis

- Das eosinophile anuläre Erythem (EAE) ist charakterisiert durch anuläre, erythematöse oder urtikarielle Läsionen mit zentrifugalem Wachstum, die Tage bis Wochen persistieren.
- Das EAE ist eine harmlose, selbstlimitierte Erkrankung unklarer Genese, die sowohl im Kindesalter als auch bei Erwachsenen auftreten kann und bei den angeführten klinischen Charakteristika als Differenzialdiagnose berücksichtigt werden sollte.
